# Pharmacokinetics of Oral Tebipenem Pivoxil Hydrobromide in Subjects with Various Degrees of Renal Impairment

**DOI:** 10.1128/aac.02407-21

**Published:** 2022-04-14

**Authors:** Gina Patel, Keith A. Rodvold, Vipul K. Gupta, Jon Bruss, Leanne Gasink, Floni Bajraktari, Yang Lei, Akash Jain, Praveen Srivastava, Angela K. Talley

**Affiliations:** a Patel Kwan Consultancy, LLC, Madison, Wisconsin, USA; b University of Illinois, Chicago, Illinois, USA; c Spero Therapeutics, Inc., Cambridge, Massachusetts, USA

**Keywords:** pharmacokinetics, renal impairment, tebipenem

## Abstract

Tebipenem pivoxil hydrobromide (TBP-PI-HBr) is an oral carbapenem prodrug antimicrobial agent with broad-spectrum activity that includes multidrug-resistant (MDR) *Enterobacterales*. This study evaluated the safety, tolerability, and pharmacokinetics of TBP-PI-HBr in healthy subjects with normal renal function (cohort 1) and subjects with various degrees of renal impairment (RI [cohorts 2 to 4]) or end-stage renal disease (ESRD) receiving hemodialysis (HD) (cohort 5). Subjects in cohorts 1 to 4 received a single oral dose of TBP-PI-HBr (600 mg). Subjects in cohort 5 received single-dose administration (600 mg) in 2 separate periods: pre-HD (period 2) and post-HD (period 1). Pharmacokinetic (PK) parameters for TBP, the active moiety, were determined using noncompartmental analysis. Compared with cohort 1, the TBP plasma area under the curve (AUC) increased 1.4- to 4.5-fold among cohorts 2 to 4, the maximum concentration of drug in plasma (*C*_max_) increased up to 1.3-fold and renal clearance (CL_R_) decreased from 13.4 L/h to 2.4 L/h as the severity of RI increased. Plasma TBP concentrations decreased over 8 to 12 h in cohorts 1 to 4, and apparent total body clearance (CL/F) correlated (*R*^2^ = 0.585) with creatinine clearance (CL_CR_). TBP urinary excretion ranged from 38% to 64% of the administered dose for cohorts 1 to 4. Subjects in cohort 5 had an approximately 7-fold increase in TBP AUC and elimination half-life (*t*_1/2_) versus cohort 1. After 4 h of HD, mean TBP plasma exposure decreased by approximately 40%. Overall, TBP plasma exposure increased with increasing RI, highlighting the renal route importance in TBP elimination. A dose reduction of TBP-PI-HBr may be needed in patients with RI (CL_CR_ of ≤50 mL/min) and those with ESRD on HD. TBP-PI-HBr was well tolerated across all cohorts. (This study has been registered at ClinicalTrials.gov under registration no. NCT04178577.).

## INTRODUCTION

The prodrug tebipenem pivoxil hydrobromide (TBP-PI-HBr) is an oral carbapenem antimicrobial that is rapidly converted to TBP, the active moiety, *in vivo* (1, 2). TBP has broad-spectrum activity and is active against both Gram-positive and Gram-negative organisms, similar to those of other carbapenems, which includes extended-spectrum β-lactamase (ESBL)-producing and multidrug-resistant (MDR) *Enterobacterales* pathogens resistant to other antibiotic classes, including fluoroquinolones (3–6). TBP has demonstrated efficacy against ESBL-producing organisms in animal infection models, including the murine neutropenic thigh infection model and the murine ascending urinary tract infection (UTI) model (7, 8). Like most β-lactams, TBP exhibits time-dependent pharmacodynamics (PD), which was observed in the thigh infection model (7). Greater efficacy with TBP was observed from dosing every 8 h (q8h). The time-dependent PD have been confirmed by *in vitro* studies in the hollow fiber infection model and one-compartment models (9). The hollow fiber infection model studies demonstrate that q8h dosing exhibits rapid bacterial killing with minimal selection of resistant subpopulations (9). TBP-PI-HBr has the potential to address an important unmet medical need for a new oral therapy effective in the treatment of serious bacterial infections due to MDR Gram-negative pathogens.

Carbapenems as a class are eliminated primarily by renal excretion, and as a consequence, require dosage adjustment in patients with various degrees of renal impairment (RI) (10–13). Because oral TBP-PI-HBr is eliminated primarily by renal excretion (14), similar to other carbapenems, it is expected that dosage adjustment will be needed in patients with severe RI. The U.S. Food and Drug Administration recommends a study be conducted in healthy subjects with impaired renal function when a drug is likely to be used in patients where RI is likely to alter the pharmacokinetics (PK) of the drug and/or its active metabolites (15). Thus, the primary objective of the study was to evaluate the PK, safety, and tolerability of TBP-PI-HBr in subjects with normal renal function, subjects with various degrees of renal insufficiency, and subjects with end-stage renal disease (ESRD) receiving hemodialysis (HD).

## RESULTS

Overall, 39 subjects received at least one dose of TBP-PI-HBr and were included in the safety and PK populations. Baseline characteristics were generally similar across cohorts, with mean (standard deviation [SD]) age of 65 (8.5) years, mean (SD) body mass index (BMI) of 28.4 (4.0) kg/m^2^, and 22 (56.4%) subjects were female (Table 1).

**TABLE 1:** Baseline characteristics

Characteristic	eGFR (mL/min/1.73 m^2^)^*a*^				ESRD (*n* = 8)
	Normal (eGFR of ≥90 [*n* = 7])	Mild (eGFR of 60 to <90 [*n* = 8])	Moderate (eGFR of 30 to <60 [*n* = 8])	Severe (eGFR of <30 [*n* = 8])	
Age, yr	62 ± 5.0	69 ± 5.4	69 ± 8.8	64 ± 9.3	58 ± 7.9
Age range, yr	56–71	62–76	52–80	53–77	42–68
Female, *n* (%)	3 (42.9)	3 (37.5)	4 (50.0)	6 (75.0)	6 (75.0)
Wt, kg	79.9 ± 9.4	71.7 ± 12.2	78.3 ± 12.1	81.2 ± 8.8	98.8 ± 16.0
BMI, kg/m^2^	27.7 ± 2.4	26.7 ± 3.7	28.3 ± 3.8	27.3 ± 3.5	32.1 ± 4.6
Race, *n* (%)					
White	2 (28.6)	6 (75.0)	8 (100)	8 (100)	0
Black or African-American	5 (71.4)	2 (25.0)	0	0	8 (100)
Hispanic or Latino, *n* (%)	0	5 (62.5)	6 (75.0)	5 (62.5)	0
eGFR, mL/min/1.73 m^2^	101 ± 8.4	73 ± 5.7	47 ± 9.5	17 ± 8.5	NA
CL_CR_, mL/min	102.6 ± 10.4	70.5 ± 12.2	55.4 ± 20.2	24.6 ± 12.5	NA

a Results for age, weight, body mass index (BMI), estimated glomerular filtration rate (eGFR), and creatinine clearance (CL_CR_) are shown as mean ± standard deviation. ESRD, end-stage renal disease; NA, not applicable.

### Pharmacokinetics. (i) Plasma.

**FIG 1:**
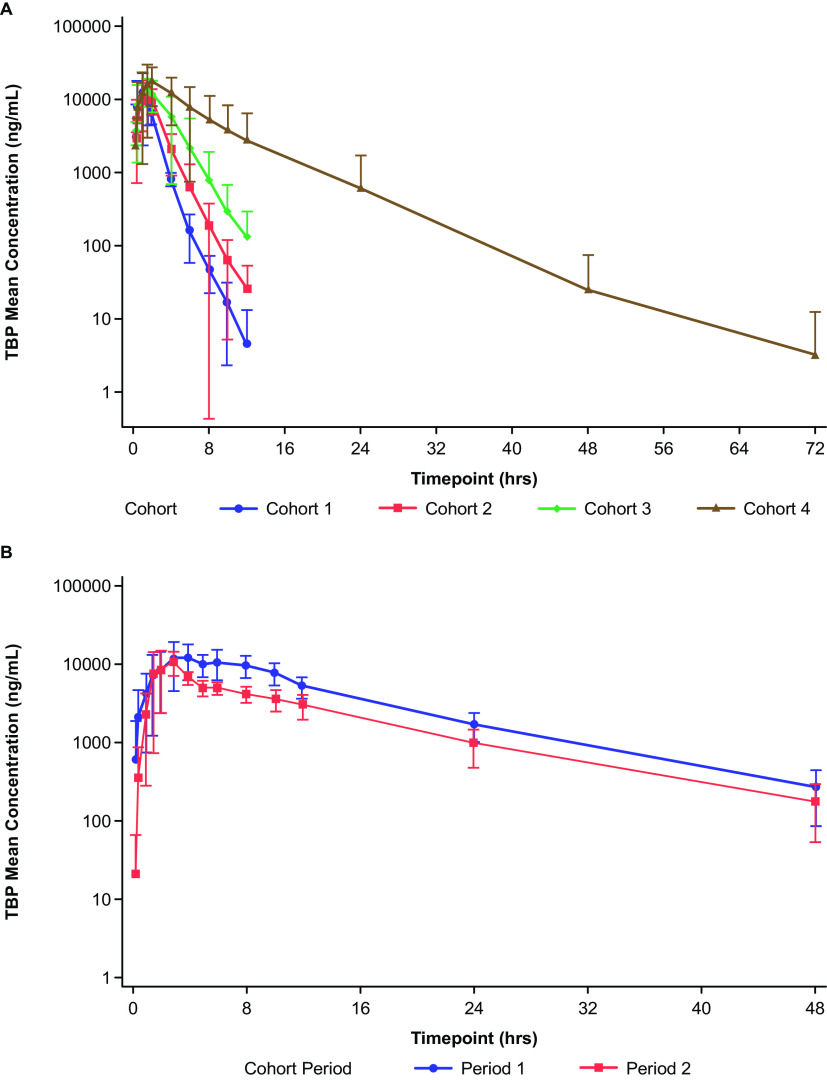
Plasma concentrations over time for TBP for cohorts 1 to 4 (A) and cohort 5 (B).

**FIG 2:**
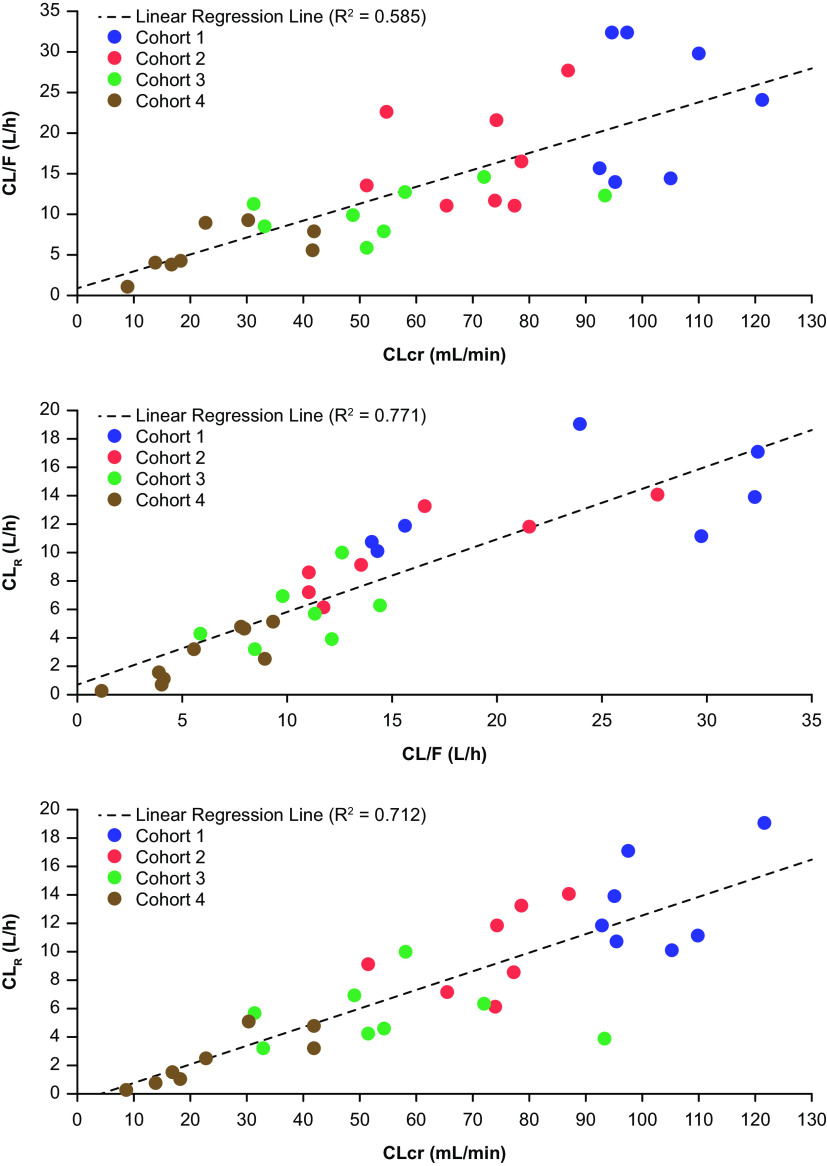
(Top) Apparent total body clearance versus estimated creatinine clearance, (middle) total body clearance versus renal clearance, and (bottom) renal clearance versus creatinine clearance of TBP for cohorts 1 to 4.

For cohorts 1 to 4 (see Materials and Methods), mean plasma TBP concentrations over time reached a peak within approximately 1.5 h and then declined over 8 to 12 h (Fig. 1). For cohorts 1 to 3, TBP plasma TBP concentrations were not measurable after 16 h. In cohort 4 with severe RI, plasma concentrations were still measurable at 72 h postdose. Elimination half-life (*t*_1/2_) and area under the curve (AUC) increased and apparent total body clearance (CL/F) decreased with increasing RI (Table 2). Apparent CL/F correlated (*R*^2^ = 0.585) with creatinine clearance (CL_CR_) for cohorts 1 to 4 (Fig. 2). Further, a correlation (*R*^2^ = 0.771 and 0.712) existed between renal clearance (CL_R_) and CL/F and CL_R_ and CL_CR_ (Fig. 2). Cohort 4 (severe RI) displayed the widest range of plasma TBP concentration-time profiles. At a CL_CR_ of <20 mL/min, the apparent CL/F of TBP was lower (mean, 3.3 L/h) than those of subjects with a CL_CR_ of ≥20 mL/min (mean, 8.0 L/h) (Table 3). In addition, TBP *t*_1/2_ and AUC from 0 h to infinity (AUC_0–inf_) were prolonged in subjects with a CL_CR_ of <20 mL/min compared to those in subjects with a CL_CR_ of ≥20 mL/min.

**TABLE 2:** TBP pharmacokinetic parameters

Parameter	Result for^*a*^:					
	Cohort 1 (normal [*n* = 7])	Cohort 2 (mild RI [*n* = 8])	Cohort 3 (moderate RI [*n* = 8])	Cohort 4 (severe RI [*n* = 8])	Cohort 5	
					Period 1 (*n* = 8)	Period 2 (*n* = 8)
*C*_max_ (μg/mL)	14.1 (67.1)	14.8 (34.0)	18.4 (14.9)	18.8 (50.8)	14.0 (50.4)	11.7 (39.1)
*T*_max_ (h)	1.5 (0.5, 2.0)	1.5 (1.0, 2.0)	1.5 (0.5, 4.0)	1.5 (1.0, 4.0)	4.0 (1.5, 6.0)	3.0 (1.5, 3.0)
AUC_0–last_ (μg · h/mL)	21.1 (40.2)	28.8 (37.1)	46.0 (29.4)	95.6 (75.4)	149 (35.6)	90.8 (16.3)
AUC_0–∞_ (μg · h/mL)	21.2 (40.2)	28.8 (37.1)	46.2 (29.9)	95.8 (75.3)	152 (35.6)	93.0 (17.4)
*t*_1/2_ (h)	1.1 ± 0.23	1.2 ± 0.14	1.3 ± 0.15	3.6 ± 1.8	7.9 ± 1.9	8.0 ± 2.1
CL/F (L/h)	21.9 (40.2)	16.1 (37.1)	10.0 (29.9)	4.83 (75.3)	3.04 (35.6)	4.97 (17.4)
*V_Z_*/F (L)	33.3 (30.0)	27.4 (36.2)	19.0 (26.7)	21.8 (45.0)	33.5 (44.2)	55.0 (22.7)

a The geometric mean (% coefficient of variation) is presented for *C*_max_, AUC_0–last_, AUC_0–∞_, CL/F and *V_Z_*/F. The arithmetic mean ± SD is presented for *t*_1/2_. The median (minimum, maximum) is reported for *T*_max_.

**TABLE 3:** TBP PK parameters for cohort 4 subjects with estimated creatinine clearances of <20 mL/min and ≥20 mL/min

PK parameter	Result for cohort 4^*a*^	
	CL_CR_ of <20 mL/min (*n* = 4)	CL_CR_ of ≥20 mL/min (*n* = 4)
AUC_0–∞_ (μg · h/mL)	183.0 (111.0, 389.0)	60.5 (49.4, 82.8)
*t*_1/2_ (h)	5.1 (4.2, 6.5)	2.0 (1.5, 2.6)
CL/F (L/h)	3.3 (1.2, 4.2)	8.0 (5.6, 9.4)
CL_R_ (L/h)	0.9 (0.3, 1.5)	3.9 (2.5, 5.1)

a The arithmetic mean (minimum, maximum) is presented. CL_CR_, estimated creatinine clearance.

For cohort 5 (ESRD), mean plasma TBP concentrations were higher post-HD (period 1) relative to pre-HD (period 2) and measurable for up to 48 h (Fig. 1). Compared with cohorts 1 and 2, the TBP maximum concentration in plasma (*C*_max_) was comparable in cohort 5 (1.3-fold higher), but the *t*_1/2_, time to *C*_max_ (*T*_max_), and AUC were 2- to 7-fold higher, and CL/F was markedly lower in both period 1 (post-dialysis) and period 2 (pre-dialysis) (Table 2). Compared to dosing in period 1, geometric mean plasma CL/F and apparent volume of distribution (*V_z_*/F) of TBP in period 2 increased, and *C*_max_ and AUC_0–∞_ decreased. Elimination *t*_1/2_ values in periods 1 and 2 were similar, and median *T*_max_ values were 4 h and 3 h in periods 1 and 2, respectively. During HD, mean ± SD values for extraction ratio (ER) and estimated HD clearance were 41.1% ± 3.7% and 8.6 ± 0.78 L/h, respectively.

All cohorts with RI exhibited higher exposure to TBP than healthy subjects (cohort 1), compared using an analysis of variance (ANOVA) model on log-transformed values of the *C*_max_, AUC from 0 h to the time of the last quantifiable concentration (AUC_0–last_), and AUC_0–∞_. Compared to healthy subjects, geometric least squares mean ratios of AUC_0–∞_ for TBP were approximately 1.4, 2.2, and 4.5 times higher in cohorts 2, 3, and 4, respectively. *C*_max_ was approximately 1.3 times higher for cohorts 3 and 4 than that for healthy subjects (cohort 1). In cohort 5, after a 4-h HD session, TBP exposure (ANOVA of log-transformed AUC_0–∞_) decreased from 152.0 μg · h/mL (period 1) to 92.8 μg · h/mL (period 2), a mean decrease of approximately 41%. A slight decrease in *C*_max_ from 14.7 μg/mL to 12.3 μg/mL was observed after a 4-h HD session. These results indicate that HD was effective in clearing TBP from the systemic circulation.

### (ii) Urine.

**FIG 3:**
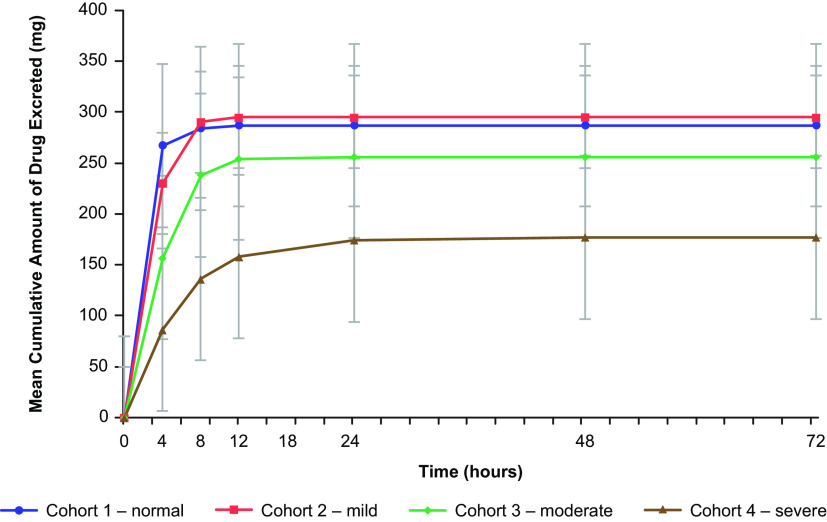
Mean (±SD) cumulative amount of TBP excreted unchanged in urine for cohorts 1 to 4.

Approximately 55% to 64% of the TBP equivalent administered dose of 462.4 mg was excreted in the urine for cohorts 1 through 3—mostly within the first 8 h postdose (Fig. 3). In comparison, subjects with severe renal impairment (cohort 4) had 38.3% of the TBP equivalent administered dose excreted in the urine. TBP renal clearance decreased as renal impairment increased, with the mean CL_R_ values ranging from 13.4 L/h to 2.4 L/h (Table 4). For cohort 4, CL_R_ was lower in the 4 subjects with CL_CR_ of <20 mL/min (*n* = 4; mean, 0.91 L/h; range, 0.27 to 1.53 L/h) compared to subjects with CL_CR_ of ≥20 mL/min (*n* = 4; mean, 3.9 L/h; range, 2.5 to 5.1 L/h).

**TABLE 4:** Excretion of TBP in urine and renal clearance for cohorts 1 to 4

PK parameter^*a*^	Result for:			
	Cohort 1 (normal [*n* = 7])	Cohort 2 (mild RI [*n* = 7])^*b*^	Cohort 3 (moderate RI [*n* = 8])	Cohort 4 (severe RI [*n* = 8])
Ae%	62.2 ± 17.5	63.9 ± 11.9	55.4 ± 17.0	38.3 ± 16.9
Ae_u_ (mg)	288 ± 81.0	295 ± 55.0	256 ± 78.7	177 ± 77.9
CL_R_ (L/h)	13.4 ± 3.44	10.0 ± 3.06	5.63 ± 2.15	2.40 ± 1.82

a Ae%, percentage excreted of TBP equivalent dose administered; Ae_u_, cumulative amount of drug excreted in urine.

b One subject in cohort 2 did not have a urine sample available during the first collection period (0 to 4 h postdose).

### Safety/tolerability.

TBP-PI-HBr was well tolerated, with 4 (10.3%) subjects reporting a total of 5 treatment emergent adverse events (TEAEs); all TEAEs were mild in severity, transient, and resolved without intervention (Table 5). Two AEs (diarrhea and abdominal pain) reported for one subject in cohort 4 were considered probably or possibly related to the study drug, respectively. No serious AEs or deaths occurred. No clinically significant abnormalities in the clinical laboratory results, electrocardiogram (ECG) findings, or physical examination were observed.

**TABLE 5:** Incidence of adverse events occurring in each cohort

AE^*a*^	No. (%) of subjects with AE				
	eGFR				ESRD (*n* = 8)
	Normal (*n* = 7)	Mild (*n* = 8)	Moderate (*n* = 8)	Severe (*n* = 8)	
Any treatment emergent AE	1 (14.3)	0	0	2 (25.0)	1 (12.5)
Any treatment-related AE	0	0	0	1 (12.5)	0
Abdominal pain	0	0	0	1 (12.5)	0
Arteriovenous fistula	0	0	0	0	1 (12.5)
COVID-19	1 (14.3)	0	0	0	0
Diarrhea	0	0	0	1 (12.5)	0
Presyncope	0	0	0	1 (12.5)	0

a AE, adverse event.

## DISCUSSION

This phase 1 study evaluated the safety, tolerability, and PK of TBP-PI-HBr in healthy subjects with normal renal function and subjects with various degrees of renal insufficiency and/or ESRD receiving HD.

TBP plasma exposure (AUC) increased significantly in subjects with RI compared to healthy subjects, while the *C*_max_ was approximately 1.3 times higher for subjects with moderate or severe RI compared to healthy subjects. CL_R_ represented approximately 50% to 58% of CL/F, and CL_R_ decreased as RI increased. Subjects with ESRD on HD had approximately a 7-fold increase in AUC and elimination *t*_1/2_ for TBP compared to those with normal renal function, although *C*_max_ values were comparable in subjects with ESRD and healthy subjects. These results indicated that renal impairment had minimal impact on TBP *C*_max_, while TBP plasma AUC increased with increasing RI due to decreased CL_R_ of TBP. Hemodialysis was effective in removing TBP from the circulation, with TBP exposure decreasing by approximately 41% after a HD session. TBP-PI-HBr appeared to be safe and well tolerated regardless of the degree of RI across subjects in this study.

Following a single 600-mg dose of TBP-PI-HBr, TBP *T*_max_ and *t*_1/2_ in subjects with normal renal function were consistent with those previously described (14). However, in the current study, *C*_max_ and AUC_0–∞_ were approximately 2-fold higher, and CL/F was approximately half of that reported in the earlier study. The increased exposure (AUC and *C*_max_) in the current study could be attributed to reduced renal clearance, which is often associated with advanced age, as age-matched subjects were recruited in cohort 1. The mean age of subjects in the healthy subject group in this study was 62 years, compared with 27 years in the earlier study (14).

The results observed with TBP-PI-HBr in subjects with various degrees of RI are consistent with those from other carbapenems, including doripenem, ertapenem, imipenem, and meropenem, where dosage modification is recommended for patients with severe or even moderate RI (12, 16–19). Studies with each of these drugs in subjects with RI found a prolonged half-life, decreased clearance, and increased exposure with increasing severity of RI (12, 16–19). As a result, dosage adjustments are recommended in patients with a CL_CR_ of ≤50 with doripenem and meropenem, a CL_CR_ of ≤30 or ESRD with ertapenem, or a CL_CR_ of ≤70 or ESRD with imipenem.

In summary, the study results characterized PK, safety, and tolerability of TBP-PI-HBr in subjects with various degrees of RI. TBP plasma AUC increased with decrease in renal function. Based on these results, a reduced dosage of TBP-PI-HBr may be needed in patients with severe RI and in patients with ESRD on HD. Population pharmacokinetic modeling of these data, along with other studies in healthy subjects and infected patients, will be used to develop dosing recommendations for patients with various degrees of renal impairment, including ESRD on HD. The safety and tolerability profile of TBP-PI-HBr was not impacted by the degree of RI in this population of otherwise healthy subjects.

## MATERIALS AND METHODS

The study was conducted between December 2019 and September 2020 at the Division of Clinical Pharmacology at University of Miami, FL, and Orlando Clinical Research Center, Orlando, FL, in accordance with the U.S. Code of Federal Regulations and ethical principles of the Declaration of Helsinki, Good Clinical Practices, and the International Council for Harmonisation guidelines. The study protocol and all amendments were reviewed by the institutional review boards for the two study centers (IntegReview IRB and University of Miami IRB). Informed consent was obtained from each subject in writing before any study procedures were performed. This study was registered at ClinicalTrials.gov under registration no. NCT04178577.

### Study design.

**FIG 4:**
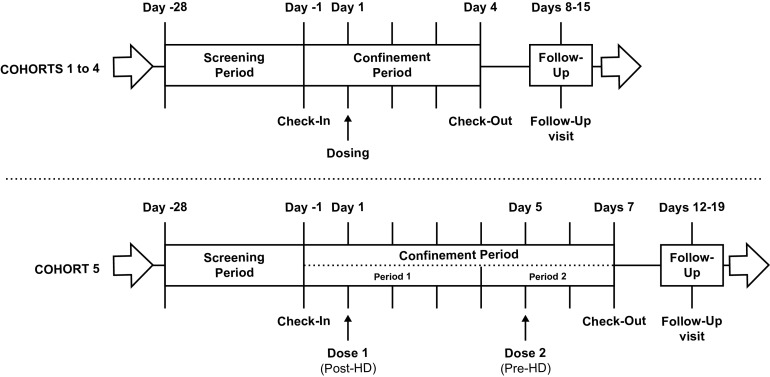
Study schematic.

This was a phase 1, multicenter, open-label study to assess the PK, safety, and tolerability of a single 600-mg oral dose of TBP-PI-HBr administered to adults with normal renal function and those with various degrees of renal insufficiency, including subjects with ESRD receiving HD (Fig. 4). Subjects were screened within 28 days prior to dosing. Study drug administration occurred on day 1 for cohorts 1 to 4 (normal, mild, moderate, or severe RI) and on days 1 and 5 for cohort 5 (ESRD on HD). Subjects remained confined to the clinical study unit from day −1 (1 day prior to study drug administration) until the completion of scheduled study procedures at the end of confinement on day 4 (cohorts 1 to 4) or on day 7 (cohort 5). A follow-up safety visit occurred 7 to 14 calendar days after the last dosing for all subjects.

Subjects were categorized into cohorts 1 to 4 at screening using the estimated glomerular filtration rate (eGFR) calculated with the Modification of Diet in Renal Disease (MDRD) (20) equation (Table 1). Subjects with ESRD receiving HD were assigned to cohort 5 at screening. Estimated creatine clearance (CL_CR_) using the Cockcroft-Gault equation (21) was calculated for subjects in cohorts 1 to 4 at screening. Initially, the study allowed simultaneous enrollment in cohorts 2 to 5. After enrollment of at least 50% of subjects in cohorts 3 and 4, matched controls were enrolled in cohort 1.

On the morning of day 1, subjects in cohorts 1 to 4 received a single dose of oral TBP-PI-HBr (600 mg [two 300-mg tablets]) with 120 mL of water. Subjects in cohort 5 received a single dose of oral TBP-PI-HBr (600 mg [two 300-mg tablets]) within 2 ± 1 h after completion of regularly scheduled HD on day 1 (period 1) and a second dose approximately 1 h prior to their regularly scheduled HD on day 5 (period 2).

### Subject selection.

Adult men or women at least 18 years of age were eligible if they had a body mass index (BMI) of ≥18.5 and ≤39.9 kg/m^2^ and body weight between 50.0 and 130.0 kg inclusive. Subjects had to be medically healthy without clinically significant abnormalities (cohort 1 only) or medically stable without clinically significant acute or chronic illness (cohorts 2 to 5) that could impact the assessment of PK and safety based on screening medical history, physical examination, vital signs, 12-lead electrocardiogram (ECG), and clinical laboratory testing. Subjects in cohort 1 had normal renal function (eGFR of ≥90 mL/min/1.73 m^2^), those in cohort 2 had an eGFR of 60 to <90 mL/min/1.73 m^2^, those in cohort 3 had an eGFR of 30 to <60 mL/min/1.73 m^2^, and those in cohort 4 had an eGFR of <30 mL/min/1.73 m^2^. Subjects in cohort 5 with ESRD were receiving HD at least 3 times per week for at least 3 months at screening. Women were nonpregnant and nonlactating, and if not postmenopausal, they were required to use an acceptable form of contraception throughout the study and for 30 days after completion.

Matching of controls was based on a rolling pooled mean for body mass index (BMI) (± 20%), sex (similar ratio of 1:1 ± 1), and mean age (±10 years) observed in subjects in cohorts 3 and 4. A minimum of 2 subjects in cohort 1 and a minimum of 6 subjects in cohorts 2 to 4 collectively were to be ≥65 years of age.

### Study assessments.

Study assessments included complete physical examinations, vital, 12-lead ECG, clinical laboratory tests (hematology, biochemistry, coagulation, and urinalysis), monitoring of adverse events (AEs), and PK samplings.

Subjects in cohorts 1 to 4 had serial blood samples collected to determine plasma TBP concentrations at predose (0 h) and 0.25, 0.5, 1, 1.5, 2, 4, 6, 8, 10, 12, 24, 48, and 72 h postdose. Total voided urine was collected over a 2-h interval from −2 to 0 h (predose) and at 0 to 4, 4 to 8, 8 to 12, 12 to 24, 24 to 48, and 48 to 72 h postdose. For subjects in cohort 5, blood samples were collected on dosing days 1 and 5 predose (0 h) and 0.25, 0.5, 1, 1.5, 2, 3, 4, 5, 6, 8, 10, 12, 24, and 48 h after dosing. On day 5 (period 2), the 1-h postdose sample was collected immediately prior to dialysis, and the 5-h postdose sample was collected immediately after the end of dialysis. The 48-h postdose samples on days 3 and 7 were collected prior to the next dialysis session if scheduled on the same day. In period 2, blood samples were collected from both the inflow (arterial) and outflow (venous) lines predialysis and at approximately 1, 2, 3, and 4 h after initiation of HD on day 5. If the HD session was shorter or longer than 4 h after the start of HD, a final sample was collected at the end of dialysis. Whole-blood samples were assayed for TBP using a validated liquid chromatography tandem mass spectrometry (LC-MS/MS) method (Charles River Laboratories, Shrewsbury, MA). The lower limit of quantitation for TBP was <0.0072 μg/mL (22). TBP blood concentrations were converted to TBP plasma concentration by correcting for the addition of (1:1) isopropyl alcohol to the sample collected and for plasmatocrit (using an average value of 55%), resulting in a multiplication factor of 3.6.

### Pharmacokinetic analysis.

For all cohorts, the following PK parameters were calculated using noncompartmental methods based on plasma TBP concentrations: maximum observed plasma concentration (*C*_max_), time to *C*_max_ (*T*_max_), AUC from 0 h to time of last quantifiable concentration (AUC_0–last_), AUC extrapolated to infinity (AUC_0–∞_), terminal elimination rate constant (λ*_Z_*), terminal elimination half-life (*t*_1/2_), apparent total body clearance (CL/F) and apparent volume of distribution (*V_Z_*/F). For cohorts 1 to 4, the following PK parameters were calculated using noncompartmental methods based on TBP urine concentrations: fraction of drug excreted in the urine expressed as a percentage of the TBP equivalent dose administered (Ae%), cumulative amount of drug excreted in urine (Ae_u_), and renal clearance (CL_R_). For cohort 5 (period 2), the extraction ratio (ER), estimated hemodialysis clearance (CL_HD_), and the amount of the dose removed by hemodialysis (*X*_HD_) were assessed. The ER was calculated as 100 × [(CA-CV)/CA], where CA and CV were predialyzer and postdialyzer paired drug concentrations at the arterial and venous sites. TBP clearance during HD (CL_HD_) was calculated using the following equation: CL_HD_ = *Q* × ER, where *Q* was the known blood flow through the dialyzer. The amount of TBP removed by dialysis (*X*_HD_) was estimated by multiplying the AUC during dialysis (i.e., AUC from the beginning of dialysis to end of dialysis [AUC_on-HD_]) by CL_HD_: *X*_HD_ = AUC_on-HD_ × CL_HD_. Under the assumptions that conversion to TBP is 100% and is instantaneous at time zero, dose-dependent PK parameters such as CL/F and *V_Z_*/F were calculated for TBP in terms of TBP-PI-HBr equivalents (i.e., 600 mg of TBP-PI-HBr = 462.37 mg of TBP). All PK evaluations were performed using Phoenix 64 WinNonlin version 8.2 (Pharsight Corporation, Mountain View, CA, USA).

### Statistical analysis.

A sample size of 8 subjects per cohort was considered sufficient to provide adequate data for inclusion into a PK analysis (15). Eight subjects were recruited in each cohort to ensure at least 6 evaluable subjects per cohort. Subjects who did not complete critical study procedures could be replaced.

Mean and individual plasma concentration-time curves were tabulated for each cohort (and for cohort 5 subjects by period). Pharmacokinetic parameters were determined for each subject and summarized by cohort (and for cohort 5 subjects by period) using descriptive statistics (arithmetic means, SD, coefficients of variation, sample size [*n*], minimum, maximum, and median). In addition, geometric means were calculated for AUC and *C*_max_.

For estimation of PK parameters for RI subjects (cohorts 2 to 5) compared to healthy subjects with normal renal function (cohort 1), an analysis of variance (ANOVA) model was used with log-transformed values of AUC_0–last_, AUC_0–∝_, *C*_max_, and CL/F as the response variables and with the fixed-effect term of cohort as a categorical variable. The estimated mean difference and associated 90% confidence interval (CI) were calculated for each RI group (versus healthy subjects) and were then back-transformed to provide geometric mean ratios and 90% CIs for each comparison. In addition, periods 1 and 2 for cohort 5 were compared to assess the effect of HD. For this analysis, data for cohort 5 from both period 1 and period 2 (before HD) were used and were considered separately in the categorical analysis.

To evaluate the effect of dialysis on TBP, log-transformed PK parameters (AUC_0–last_, AUC_0–∝_, *C*_max_, and CL/F) obtained with dosing before HD (test) versus dosing after HD (reference) in ESRD subjects were evaluated using an ANOVA model with period as the factor, body weight at the baseline, age, and sex as covariates, and subject as the random effect. A two-sided 90% CI for the estimated ratio of the effect of dialysis (using the period effect) was calculated for all PK parameters (AUC_0–last_, AUC_0–∝_, *C*_max_, and CL/F). The ratio of the geometric means and their CI was obtained by back-transforming the estimated mean difference and its corresponding CI. All statistical evaluations were conducted using SAS version 9.1.3.
